# The oral bacterial microbiota facilitates the stratification for ulcerative colitis patients with oral ulcers

**DOI:** 10.1186/s12941-023-00646-3

**Published:** 2023-11-09

**Authors:** Jun Xu, Yu Zhang, Xiao-Hui Fang, Yun Liu, Yi-Bo Huang, Zi-Liang Ke, Yang Wang, Yi-Fan Zhang, Yang Zhang, Jian-Hua Zhou, Hui-Ting Su, Ning Chen, Yu-Lan Liu

**Affiliations:** 1 Department of Gastroenterology, Peking University People’s Hospital, No.11, Xizhimen South Street, Xicheng District, Beijing, 100044 China; 2 Clinical Center of Immune-Mediated Digestive Diseases, Peking University People’s Hospital, No. 11, Xizhimen South Street, Xicheng District, Beijing, 100044 China; 3 Institute of Clinical Molecular Biology and Central Laboratory, Peking University People’s Hospital, No. 11, Xizhimen South Street, Xicheng District, Beijing, 100044 China

**Keywords:** Ulcerative colitis, Oral ulcer, Bacterial microbiota, Response, Stratification

## Abstract

**Background:**

Clinically, a large part of inflammatory bowel disease (IBD) patients is complicated by oral lesions. Although previous studies proved oral microbial dysbiosis in IBD patients, the bacterial community in the gastrointestinal (GI) tract of those IBD patients combined with oral ulcers has not been profiled yet.

**Methods:**

In this study, we enrolled four groups of subjects, including healthy controls (CON), oral ulcer patients (OU), and ulcerative colitis patients with (UC_OU) and without (UC) oral ulcers. Bio-samples from three GI niches containing salivary, buccal, and fecal samples, were collected for 16S rRNA V3-V4 region sequencing. Bacterial abundance and related bio-functions were compared, and data showed that the fecal microbiota was more potent than salivary and buccal microbes in shaping the host immune system. ~ 22 UC and 10 UC_OU 5-aminosalicylate (5-ASA) routine treated patients were followed-up for six months; according to their treatment response (a decrease in the endoscopic Mayo score), they were further sub-grouped as responding and non-responding patients.

**Results:**

We found those UC patients complicated with oral ulcers presented weaker treatment response, and three oral bacterial genera, i.e., *Fusobacterium*, *Oribacterium*, and *Campylobacter*, might be connected with treatment responding. Additionally, the salivary microbiome could be an indicator of treatment responding in 5-ASA routine treatment rather than buccal or fecal ones.

**Conclusions:**

The fecal microbiota had a strong effect on the host’s immune indices, while the oral bacterial microbiota could help stratification for ulcerative colitis patients with oral ulcers. Additionally, the oral microbiota had the potential role in reflecting the treatment response of UC patients. Three oral bacteria genera (*Fusobacterium*, *Oribacterium*, and *Campylobacter*) might be involved in UC patients with oral ulcers lacking treatment responses, and monitoring oral microbiota may be meaningful in assessing the therapeutic response in UC patients.

**Supplementary Information:**

The online version contains supplementary material available at 10.1186/s12941-023-00646-3.

## Background

Ulcerative colitis (UC) is a chronic inflammatory disease mainly involving the colon; its pathogenesis is multifactorial, including genomic risks, immunological dysfunction, environmental factors, and gut microbial dysbiosis [1]. Increasing evidence has shown that microbial-host interaction is pivotal in homeostasis and pathogenesis. The gut microbiome educates the immune system during host development. Those germ-free animal models have immunological defects in the intestine, including smaller lymph nodes and Peyer’s patches decreased the number of the helper T cells [2]. Besides, gut microbial dysbiosis is also involved in the pathogenesis of various gut and even extra-intestinal diseases. The gut-liver [3], gut-brain [4], gut-lung [5], gut-bone marrow [6], and gut-skin axes [7] based etiologies have explained the underlying mechanisms of multi-organ diseases, such as metabolic associated fatty liver disease (MAFLD), autism spectrum disorder, chronic obstructive pulmonary disease (COPD), arthritis, and atopic dermatitis, etc.

Notably, ~ 2–34% of UC patients are complicated with oral manifestations, such as ulceration [8]. Interestingly, the oral microbiome has also been reported to be correlated with IBD [9–11], which indicates that the oral microbial community has the potential to trigger in situ lesions in IBD patients. Nevertheless, the microbial community in UC patients with oral ulcers has not been profiled.

To recover the microbial community in disorder, fecal microbiota transplantation (FMT) tends to be an efficient therapeutic strategy for some diseases [12]. Our previous studies have also reported that the therapeutic factors in 5-ASA routine treatment drive microbial alteration, whether in bacterial or fungal communities [13, 14]. These studies showed that the microbes-based clinical management is reasonable; however, it is unclear whether it would be meaningful for monitoring microbial profiles in discriminating disease activities and treatment efficacy. Our previous study showed that the *Escherichia-Shigella* richness in inflamed mucosa positively correlates with the UC activity [13]. Also, microbial contents have been used to predict activity in Crohn’s disease [15], irritable bowel syndrome [16], and other diseases [17, 18].

Based on these studies, to explore the bacterial community in the GI tract of IBD patients combined with oral ulcers, we analyzed the microbial profiles by 16S rRNA sequencing at three GI niches, including salivary, buccal, and fecal samples of UC patients with or without oral ulcers. We found that complicating with oral ulcers made UC patients a weak treatment response; and three oral bacterial genera, including *Fusobacterium*, *Oribacterium*, and *Campylobacter*, might be involved in the process. These results highlighted the significance of stratification of UC patients with oral diseases. Additionally, monitoring oral microbiota may be meaningful in assessing the therapeutic response in UC patients.

## Methods

### Study design and sample collection

**Fig. 1:**
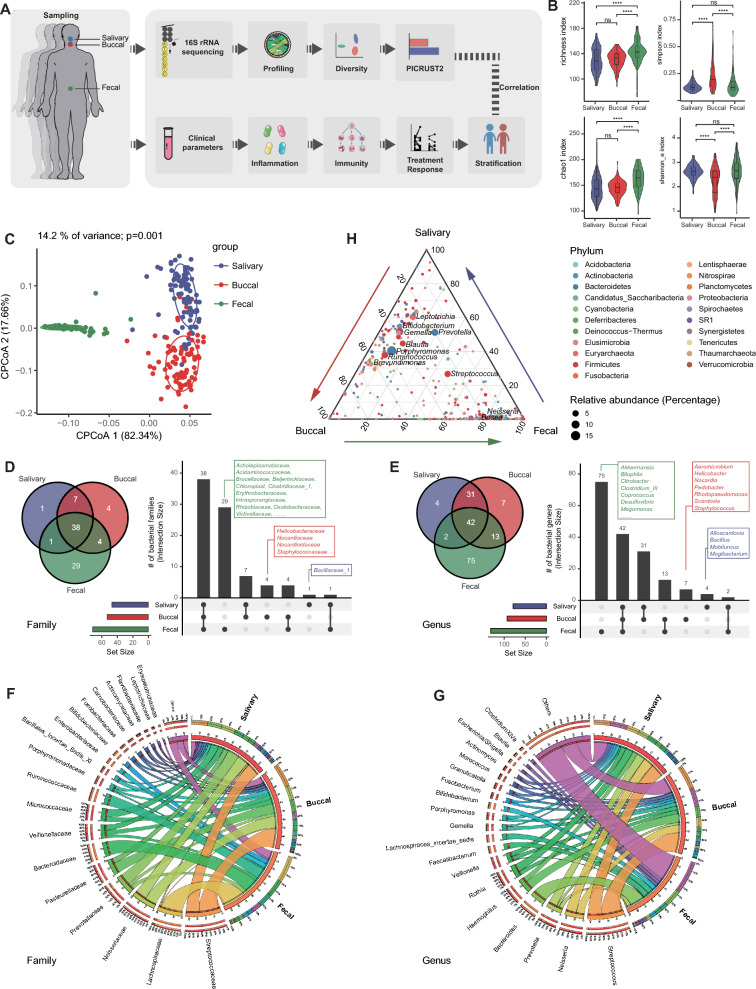
Bacterial profiles at different gastrointestinal tract niches. **A** Study design. Three types of samples from differed GI niches were collected in this study, including salivary, buccal, and fecal samples. **B** Alpha diversity indices of the microbiota, including the richness, Simpson’s, Shannon’s, and Chao1 indices. Horizontal bars within boxes represent medians. The tops and bottoms of the boxes represent the 75th and 25th percentiles, respectively. The upper and lower whiskers cover 1.5 × the interquartile range from the upper and lower edges of the box, respectively. *P*-values were obtained using the one-way ANOVA test (comparisons among four groups). **C** The constrained principal coordinate analysis based on the Bray–Curtis distance. The R software (v 4.0.1) with the *vegan* (v 2.5–7) package were used and *P*-values were obtained using permutational multivariate analysis of variance (PERMANOVA). **D** and **E** The upset plot shows the bacterial family (**D**) and genus (**E**) count in each GI niche. **F** and **G** Relative abundance of the top 20 bacterial families (**F**) and genera (**G**). Visualization was performed using Circos (http://circos.ca/). The right circle in the outer part shows the groups and relative proportions of bacterial species. The left outer ring and inner bands indicate the relative proportions (%) of bacterial genera in the different groups. The left inner circle represents the relative abundances of all bacteria. **H** The ternary plot shows the distribution of the specific bacterial genus in each GI niche. The point color represents the phylum classification of the bacterial genus. The point size presents the mean percentage of a specific genus

This study was approved by the Institutional Medical Ethics Review Board of Peking University People’s Hospital. All subjects were enrolled at Peking University People’s Hospital from January 2017 to January 2020. To test the bacterial difference in UC patients with or without oral ulcers, we enrolled four groups of subjects, including healthy controls (CON), patients with only oral ulcers (OU), UC patients without oral ulcers (UC), and UC patients with oral ulcers (UC_OU) (Fig. 1A and Table 1). The healthy subjects were recruited from volunteers taking 5-ASA routine health examinations in Peking University People’s Hospital. The exclusion criteria of healthy participants included periodontitis, OU, UC, Crohn’s disease, Behçet’s disease, and other chronic or acute inflammatory statuses with endoscopically confirmation. The diagnosis of UC was established according to the World Gastroenterology Organization Global Guidelines [19]. Patients in the OU group were confirmed to suffer only oral ulcers without other oral and systemic diseases. In addition, those UC patients enrolled in the UC_OU group were historically recurrent oral ulcers, and suffering oral ulcers during sampling. All subjects were informed to avoid taking antibiotics, yogurt, and other probiotics within 4 weeks; hereafter, all subjects’ written informed consents were obtained before sampling. In addition, we documented the demographic and clinical information of all subjects.

**Table 1:** Demographic and clinical profiles of patients from different groups and healthy controls

	CON (*n* = *28*)	OU (*n* = 18)	UC (*n* = *37*)	UC_OU (*n* = 17)
Sex, Men, No. (%)	21 (75)	8 (44.4)	22 (59.5)	10 (58.8)
Age (years)	56.82 ± 2.15	30.72 ± 3.60	42.59 ± 2.24*	35.82 ± 2.79
Height (m)	1.70 ± 0.01	1.69 ± 0.02	1.69 ± 0.01	1.69 ± 0.02
Weight (kg)	70.57 ± 1.98	58.5 ± 1.97	65.23 ± 1.78*	63.6 ± 3.42
BMI (kg/m^2^)	24.42 ± 2.68	20.44 ± 0.43	22.75 ± 0.48*	22.05 ± 0.86
Process (year)			4.28 ± 0.75	4.91 ± 1.32
Laboratory tests				
WBC (× 10^9^)	6.11 ± 0.22	6.36 ± 0.39	6.31 ± 0.26	6.37 ± 0.70
LY%	25.6 ± 1.19	34.9 ± 2.06*	26.94 ± 1.47^+^	34.73 ± 2.85***^##^
MO%	7.25 ± 0.31	7.82 ± 0.65	8.1 ± 0.31	7.64 ± 0.58
NE%	63.58 ± 1.65	54.99 ± 2.52*	61.25 ± 1.56	53.91 ± 3.29**^#^
EOS%	1.89 ± 0.35	1.61 ± 0.37	3.10 ± 0.39*	3.21 ± 0.84
BAS%	0.62 ± 0.06	0.68 ± 0.11	0.60 ± 0.04	0.51 ± 0.06
LY (× 10^9^)	1.57 ± 0.10	2.18 ± 0.13*	1.69 ± 0.11^+^	2.1 ± 0.25*^#^
MO (× 10^9^)	0.44 ± 0.02	0.49 ± 0.03	0.51 ± 0.03	0.47 ± 0.05
NE (× 10^9^)	3.95 ± 0.16	3.54 ± 0.34	3.89 ± 0.20	3.63 ± 0.67
EOS (× 10^9^)	0.12 ± 0.03	0.10 ± 0.02	0.19 ± 0.03	0.19 ± 0.05
BAS (× 10^9^)	0.03 ± 0.01	0.04 ± 0.01	0.03 ± 0.01	0.03 ± 0.01

*P < 0.05, **P < 0.01, ***P < 0.001, compared with CON

^+^*P* < 0.05, ^++^*P* < 0.01, compared with OU

^#^*P* < 0.05, ^##^*P* < 0.01, compared with UC

### Sample collection and DNA extraction

Bio-samples from three different spatial locations in the digestive tract, including salivary, buccal, and fecal samples, were collected for the following test. Briefly, The patient rinses his mouth with clean water before sampling and collects the unstimulated saliva into a sterile DNase-free and RNase-free centrifuge tube for about 5–8 min. After that, buccal mucosa was swabbed 50 times on the left and right side walls of mouth with a sterile cotton swab without touching teeth and gums. Fecal samples were collected in a Stool Collection Tube with Stool Stabilizer (German, Stratec Molecular) and stored with a – 80 °C freezer as in previous reports[13, 14]. According to the manufacturer’s instructions, the bacterial genomic DNA was extracted with the PSP Spin Stool DNA Kit (Stratec Molecular). Hereinafter, the bacterial 16S V3-V4 rRNA was amplified using 338F (5′-ACTCCTACGGGAGGCAGCAG-3′) and 806R primers (5′-GGACTACHVGGGTWTCTAAT-3′) following the protocol described in our previous study [20]. In brief, a 20-μL reaction system containing the FastPfu Polymerase (TransGen Biotech Co., Beijing, China) was used to amplify the 16S rRNA [20]. All PCR products were purified and added with sample-specific barcodes.

### 16S rRNA sequencing and bioinformatic analysis

The Illumina Miseq platform (Illumina, San Diego) was used for 16S rRNA sequencing. The raw data were produced with the Vsearch v2.8.1 [21] and Usearch v11 (bit 64) software [22]. Vsearch was performed for merging the original data, quality control, primer, and barcode sequences excision. In total, 2,317,709 sequences were removed, leaving 55,571,009 sequences for further analysis. Redundant sequences were then filtered, and 15,157,810 unique sequences were retained. Vsearch was used for discarding low-occurrence sequences. 33,920,292 sequences were removed, and 12,254 amplicon sequences were left. Remove 4951 chimers in the 12,254 sequences and 7,303 high-quality sequences were acquired. Subsequently, chimeras filtering was conducted with the amplicon sequence variants (ASVs) method, followed by sequencing error checking using Usearch as in our previous report [23, 24]. As a result, 4,121 high-qualified ASVs were obtained and aligned with Ribosomal Database Project’s (RDP) training Set 16 [25].

### Functional prediction with the PICRUSt2

The Phylogenetic Investigation of Communities by Reconstruction of Unobserved States (PICRUSt2) software was used for functional prediction [26]. In investigating the etiology of IBD, we focused on the immune-mediated pathways in these studies [27].

### The stratification of UC patients

The clinical parameters, including complement C3 (C3), complement C4 (C4), C-reactive protein (CRP), erythrocyte sedimentation rate (ESR), immunoglobulin M (IgM), immunoglobulin A (IgA), immunoglobulin G (IgG), monocyte (Mo), lymphocyte (LY), monocytes’ percentage (MO_P), white blood cell (WBC), lymphocyte’ percentage (LY_P), eosinophil (EOS), Hemoglobin(Hb), platelet (PLT), basophils(BAS), basophils’ percentage (BAS_P), neutrophils (NE), neutrophils’ percentage (NE_P), were recorded for all UC patients at the sampling baseline.

Except for a small group of UC patients losing touch, ~ 22 UC patients and 10 UC_OU patients underwent a six-month following-up. In comparing clinical parameters, especially in the Mayo clinical score, the patients were stratified into responding and non-responding groups (Fig. 1A and Table 2).

**Table 2:** Demographic and clinical profiles of subjects followed-up

	UC (*n* = 22)		UC_OU (*n* = 10)	
	Baseline	Follow up	Baseline	Follow up
WBC (× 10^9^)	6.31 ± 0.26	6.52 ± 2.53	6.37 ± 2.79	6.20 ± 1.92
LY%	26.94 ± 1.47	27.04 ± 1.52	34.73 ± 2.85	31.52 ± 2.02
MO%	8.01 ± 0.32	7.6 ± 0.35	7.64 ± 0.58	6.62 ± 1.13
NE%	61.25 ± 1.56	61.19 ± 2.08	53.91 ± 3.29	56.71 ± 3.34
EOS%	3.11 ± 0.39	3.56 ± 0.91	3.21 ± 0.84	1.56 ± 0.54
BAS%	0.60 ± 0.04	0.62 ± 0.07	0.51 ± 0.06	0.59 ± 0.09
LY (× 10^9^)	1.69 ± 0.11	1.68 ± 0.11	2.09 ± 0.25	1.92 ± 0.20
MO (× 10^9^)	0.51 ± 0.03	0.48 ± 0.04	0.47 ± 0.05	0.38 ± 0.07
NE (× 10^9^)	3.89 ± 0.20	4.07 ± 0.43	3.63 ± 0.67	3.78 ± 0.46
EOS (× 10^9^)	0.19 ± 0.03	0.25 ± 0.09	0.19 ± 0.05	0.09 ± 0.04
BAS (× 10^9^)	0.03 ± 0.01	0.03 ± 0.01	0.03 ± 0.01	0.03 ± 0.01
Mayo Scores	5.73 ± 0.48	6.46 ± 0.63	6.4 ± 1.10	6.50 ± 1.03
ESR (mm/h)	15.89 ± 2.94	19.71 ± 5.64	17.69 ± 3.70	17.43 ± 6.28
CRP (mg/L)	10.79 ± 3.10	4.39 ± 1.16	15.13 ± 7.09	8.32 ± 3.32

### Statistical analysis and data visualization

The STAMP software (v 2.1.3) [28] and R software (v 4.0.1; R Foundation for Statistical Computing, Vienna, Austria) with the *ggplot2* (v 3.3.2) package were used for data visualization [29]. Permutational multivariate analysis of variance (PERMANOVA, Adonis test of vegan v 2.5–6) was performed for statistical analysis of beta diversity. The independent sample *t*-test and nonparametric Mann–Whitney *U* test compared two groups. One-way analysis of variance (ANOVA) and Kruskal–Wallis H nonparametric tests compared the three groups. Spearman’s correlation analysis was performed, and the *P-*value was corrected with the false discovery rate (FDR). Significant correlations were visualized using the *pheatmap* package (v1.0.12). A *P*-value or FDR ≤ 0.05 was considered statistically significant.

## Results

### The microbial community composition of salivary, buccal, and fecal samples

To investigate the characteristic microbial profiles in the GI tract, we conducted the 16S rRNA sequencing and compared the bacterial diversity in different GI locations first. The alpha diversity analysis revealed that richness and chao1 indices were gradually higher in fecal samples than in salivary and buccal samples (Fig. 1B, Additional file 5: Table S1), indicating a higher microbial community richness. In addition, the Simpson index was lower, and the Shannon index was higher in salivary and fecal samples than in buccal samples (Fig. 1B, Additional file 5: Table S1), which suggested higher evenness of microbial community composition in salivary and fecal samples. Furthermore, beta diversity analysis with constrained principal coordinates analysis (CPCoA) showed an adjacency between salivary and buccal samples while a separation between fecal and oral (salivary and buccal) samples in Bray–Curtis distance. Nonetheless, there were significant differences among the three groups (Adonis *P* < 0.05, Fig. 1C, Additional file 5: Table S2). These data suggested that salivary and buccal samples share similar microbial community composition to a certain degree, whereas fecal samples did not.

Further analysis showed that thirty-eight bacterial families were shared in three different niches and oral samples shared seven other families (Fig. 1D and Additional file 5: Table S3). In addition, there were twenty-nine special bacterial families in fecal samples (*e.g.*, Acholeplasmataceae, Acidaminococcaceae, and Brucellaceae), four in buccal samples (Hicobacteraceae, Nocardiaceae, Nocardiodaceae, and Staphylococcaceae) and one unique bacterial family in salivary samples (Bacillaceae_1) (Fig. 1D and Additional file 5: Table S3). At the genus level, data showed that forty-two bacterial genera were shared among the three sites, and seventy-three in oral samples. As to unique genera, there were seventy-five bacteria specific in fecal samples (*e.g.*, *Akkermansia*, *Bilophila*, and *Citrobacter*, etc*.*), seven in buccal samples (*e.g.*, *Aeromicrobium*, *Helicobacter*, and *Nocardia*, etc*.*), while four in salivary samples (including *Alloscardovia*, *Bacillus*, *Mobiluncus*, and *Mogibacterium*) (Fig. 1E and Additional file 5: Table S3). These data indicated a higher bacterial richness in the feces, and some bacterial species were shared in oral and fecal samples.

We further analyzed the twenty most abundant bacterial families and genera and found that Streptococcaceae and Neisseriaceae mainly colonize the oral cavity at the family level, while Lachnospiraceae, Bacteroidaceae, Ruminococcaceae, and Enterobacteriaceae colonized with a high richness in feces (Fig. 1F and Additional file 5: Table S5). At the genus level, *Streptococcus*, *Neisseria*, *Haemophilus*, and *Rothia* occupied the oral cavity with high abundance; while *Faecalibacterium*, *Bacteroides*, *Bifidobacterium*, *Escherichia*/*Shigella*, and *Lachnospiraceae_incertae_sedis* were highly abundant in feces (Fig. 1G, H and Additional file 5: Table S5-6).

These results indicate that the bacteria in salivary and buccal samples are highly similar at the family and genus levels; they share many common and similar dominant bacteria but fewer unique bacteria. In contrast, there are many specific bacterial families and genera in feces but not in oral samples, indicating that it is usually difficult for most bacteria in the oral cavity to colonize in the gut. Thus, we speculate that this may be related to the different microenvironments in the oral cavity and gut.

### The salivary bacterial microbes in UC patients complicated by oral ulcers

**Fig. 2:**
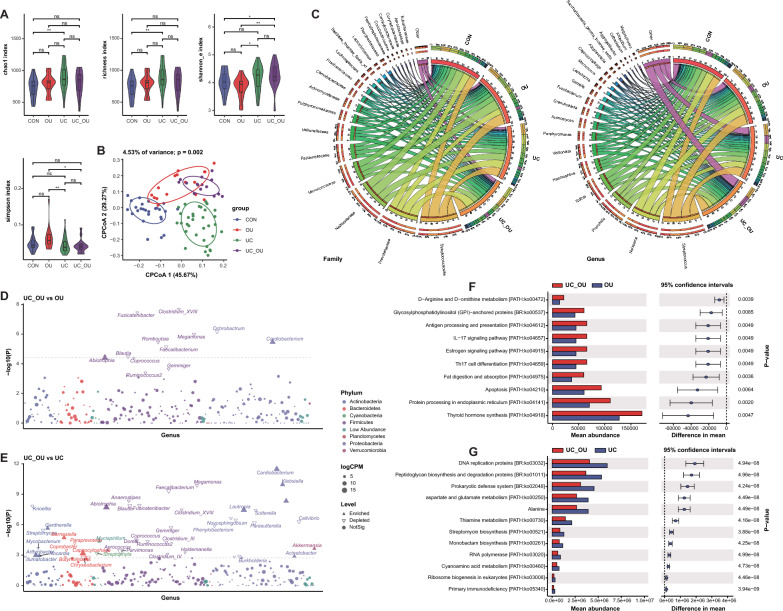
The bacterial profile in salivary samples of UC patients with or without oral ulcers. **A** Alpha diversity indices of the microbiota, including the richness, Simpson’s, Shannon’s, and Chao1 indices. Horizontal bars within boxes represent medians. The tops and bottoms of the boxes represent the 75^th^ and 25^th^ percentiles, respectively. The upper and lower whiskers cover 1.5 × the interquartile range from the upper and lower edges of the box, respectively. *P*-values were obtained using the one-way ANOVA test (comparisons among four groups). **B** The constrained principal coordinate analysis based on the Bray–Curtis distance. The R software (v 4.0.1) with the *vegan* (v 2.5–7) package were used, and *P*-values were obtained using permutational multivariate analysis of variance (PERMANOVA). **C** Relative abundance of the top 20 bacterial families (the left panel) and genera (the right panel). Visualization was performed using Circos (http://circos.ca/). The right circle in the outer part shows the groups and relative proportions of bacterial species. The left outer circle and inner bands show the relative proportions (%) of bacterial genera in the different groups. The left inner circle represents the relative abundances of all bacteria. **D** and **E** Comparative analysis of bacterial genus abundance between two groups (**D** UC_OU *vs.* OU; **E** UC_OU *vs.* UC). The EdgeR package was used for comparative analysis. The difference between the two groups is shown as a Manhattan diagram. Point shape indicates the genus enriched, depleted, or not significant in the former group compared with the latter. Point size indicates the counts of a specific genus. CPM, count per million. **F** and **G** Comparative analysis of bacterial function between two groups (**F** UC_OU *vs.* OU; **G** UC_OU *vs.* UC). Phylogenetic Investigation of Communities annotated the pathway information by Reconstruction of Unobserved States (PICRUSt2) software by referring to the Kyoto Encyclopedia of Genes and Genomes (KEGG) database. The STAMP software was used for data visualization. CON, healthy controls; OU, patients with only oral ulcers; UC, UC patients without oral ulcers; UC_OU, UC patients with oral ulcers; ns, not significant; *P-value < 0.05; **P-value < 0.01

To uncover the microbial features in salivary samples of UC_OU patients, we further profiled the salivary bacterial community in all subjects. Compared with the CON group, the richness increased in the UC group, and the diversity and evenness elevated in the UC_OU group (Fig. 2A and Additional file 5: Table S1). Moreover, the beta diversity analysis revealed that the salivary microbial community composition was significantly different between the groups of patients and the CON (Adonis *P* = 0.046 for OU, Adonis *P* = 0.002 for UC, *P* = 0.004 for UC_OU *vs.* CON, respectively, Fig. 2B, S1B, and Additional file 5: Table S2). Furthermore, the analysis of the twenty most abundant bacterial families and genera showed that there were remarkable differences in abundance between the groups of patients and the CON at the family and genus level (Fig. 2C and Additional file 5: Table S5-6). Further investigation of UC_OU and OU manifested that some bacteria decreased (*e.g.*, *Blautia*, *Clostridium_XIII*, and *Faecalibacterium*), and some increased (*e.g.*, *Abiotrophia*) (Fig. 2D and Additional file 5: Table S7). Surprisingly, there were up to fifty different genera between the UC_OU and UC groups, including the increasing *Klebsiella, Arthrobacter*, *Barnesiella*, and the decreasing *Blautia*, *Clostridium_XIII*, and *Faecalibacterium* (Fig. 2E and Additional file 5: Table S7). We also compared the groups of patients with the CON respectively, and found that some genera (*e.g.*, *Arthrobacter*, *Barnesiella*, *Alistipes*) increased and some (*e.g.*, *Mogibacterium*) decreased consistently (Additional file 1: Figure S1C-E and Additional file 5: Table S7).

Furthermore, the PICRUSt2 analysis showed that in the group of UC_OU, the immune-related signaling pathways (including antigen processing and presentation, IL-17 signaling, and Th17 cell differentiation, etc.) were active compared with the OU group. The anti-inflammatory pathway (*e.g.*, thiamine metabolism [30]) was restrained compared with the UC group (Fig. 2F, G and Additional file 5: Table S8), indicating a higher immune activation and inflammatory states in UC_OU patients.

In general, the increment of *Arthrobacte* and *Barnesiella* and the reduction of *Blautia*, *Clostridium_XIII*, and *Faecalibacterium* in UC_OU patients may be essential factors in mediating the occurrence and development of the disease by regulating immune responses and inflammatory pathways.

### The buccal bacterial community in UC patients complicated by oral ulcers

**Fig. 3:**
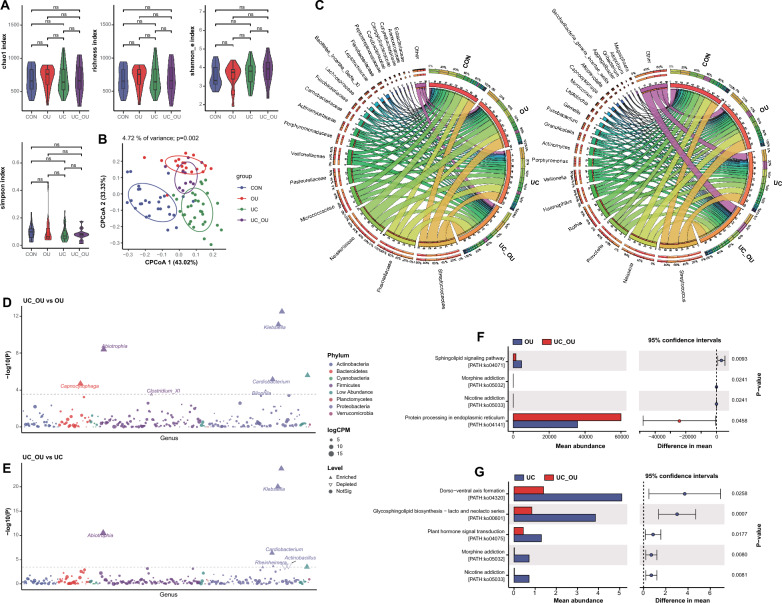
The bacterial profile in buccal samples of UC patients with or without oral ulcers. **A** Alpha diversity indices of the microbiota, including the richness, Simpson’s, Shannon’s, and Chao1 indices. Horizontal bars within boxes represent medians. The tops and bottoms of the boxes represent the 75th and 25th percentiles, respectively. The upper and lower whiskers cover 1.5 × the interquartile range from the upper and lower edges of the box, respectively. *P*-values were obtained using the one-way ANOVA test (comparisons among four groups). **B** The constrained principal coordinate analysis based on the Bray–Curtis distance. The R software (v 4.0.1) with the *vegan* (v 2.5–7) package were used, and *P*-values were obtained using permutational multivariate analysis of variance (PERMANOVA). **C** Relative abundance of the top 20 bacterial families (the left panel) and genera (the right panel). Visualization was performed using Circos (http://circos.ca/). The right circle in the outer part shows the groups and relative proportions of bacterial species. The left outer circle and inner bands show the relative proportions (%) of bacterial genera in the different groups. The left inner circle represents the relative abundances of all bacteria. **D** and **E**, Comparative analysis of bacterial genus abundance between two groups (**D** UC_OU *vs.* OU; **E** UC_OU *vs.* UC). The EdgeR package was used for comparative analysis. The difference between the two groups is shown as a Manhattan diagram. Point shape indicates the genus enriched, depleted, or not significant in the former group compared with the latter. Point size indicates the counts of a specific genus. CPM, count per million. **F** and **G**, Comparative analysis of bacterial function between two groups (**F** UC_OU *vs.* OU; **G** UC_OU *vs.* UC). Phylogenetic Investigation of Communities annotated the pathway information by Reconstruction of Unobserved States (PICRUSt2) software by referring to the Kyoto Encyclopedia of Genes and Genomes (KEGG) database. The STAMP software was used for data visualization. CON, healthy controls; OU, patients with only oral ulcers; UC, UC patients without oral ulcers; UC_OU, UC patients with oral ulcers; ns, not significant; *P-value < 0.05; **P-value < 0.01

There were no significant differences in alpha diversity among the buccal samples of the CON, OU, UC, and UC_OU. The beta diversity analysis showed that there were significant differences when the OU/UC, but not the UC_OU group, compared with the CON group (Adonis *P* = 0.008 for OU, *P* = 0.001 for UC and *P* = 0.102 for UC_OU vs. CON, respectively, Fig. 3B, S2A, and Additional file 5: Table S2).

We further investigated the buccal bacterial composition in different groups of subjects. At the family level, the top twenty bacteria in abundance levels differed not so markedly among all groups of subjects (Fig. 3C and Additional file 5: Table S5). While at the genus level, the abundance of some bacteria, such as *Neisseria* and *Rothia*, declined slightly in the UC_OU group. As for *Neisseria,* a previous study found a falling abundance of which at the inflamed site of UC patients compared with the corresponding area of non-IBD controls [31]; in addition, the abundance of *Actinobacillus* and *Fusobacterium* varied primarily among these groups (Fig. 3C and Additional file 5: Table S6).

Using further differential analysis, we found that, compared with the CON group, the variation of bacteria was considerably conspicuous among OU, UC, and UC_OU group at the genus level; for instance, the abundance of *Barnesiella*, *Alistipes*, and *Rhodopseudomonas* ascended, while *Actinobacillus* descended (Additional file 2: Figure S2B-D and Additional file 5: Table S9). Then, we compared oral bacterial richness between UC_OU and OU groups and found very few differential bacteria, which manifested the increase of *Abiotrophia, Cardiobacterium*, and *Klebsiella* (Fig. 3D and Additional file 5: Table S9). Interestingly, *Abiotrophia defectiva* was related to pro-inflammatory response in the oral cavity [32]; moreover, *Klebsiella pneumoniae* was reported to aggravate chronic intestinal inflammation by destructing the intestinal epithelial barrier [33]. In contrast with UC patients, there was a marked increase in the abundance of *Abiotrophia, Cardiobacterium,* and *Klebsiella* in the UC_OU patients; on the contrary, the abundance of *Actinobacillus* decreased notably (Fig. 3E and Additional file 5: Table S9). Hence, we inferred that these bacteria probably contributed to oral ulcers in the UC_OU patients.

A previous study found that decreased sphingolipids correlated with gut inflammation in IBD subjects [34]. Our PICRUSt2 analysis also observed suppressed sphingolipid signaling pathway in the UC_OU patients compared with OU patients. In addition, there was an enrichment of protein processing in the endoplasmic reticulum in the UC_OU patients (*vs.* OU, Fig. 3F and Additional file 5: Table S10). It was found multiple immune cells could activate that unfolded protein response at distinct levels [35]. Furthermore, the differences in signal pathways between UC and UC_OU patients showed that the expression level of some signal pathways declined, such as dorso-ventral axis formation and glycosphingolipid biosynthesis (Fig. 3G and Additional file 5: Table S10); among which glycosphingolipid was reported to correlate with regulating immune signaling with facilitating bacterial entering host cells [36]. The comparison results between OU and CON patients unveiled that some pathways diminished in the OU patients, such as pyrimidine metabolism, RNA polymerase, protein phosphatases and associated proteins, and secondary bile acid biosynthesis (Additional file 2: Figure S2E and Additional file 5: Table S10), which were all associated with immunity [37–39]. Moreover, there was an enriched apoptosis pathway in the UC group compared with the CON one (Additional file 2: Figure S2F and Additional file 5: Table S10), which was paralleled with a previous review that showed the pathogenic function of caspase-mediated intestinal epithelial cell apoptosis in the IBD [40].

In brief, buccal bacterial features in UC_OU subjects differed from UC alone or OU. and the abundance of some bacteria (such as *Abiotrophia*, and *Klebsiella*) in the buccal mucosa may help to distinguish between UC_OU subjects and UC alone or OU. Furthermore, the alterations of some signaling pathways related to immune cells or processes implied that immune factors might participate in the occurrence and development of oral ulcers in UC_OU patients.

### The fecal bacterial microbiota in UC_OU patients

**Fig. 4:**
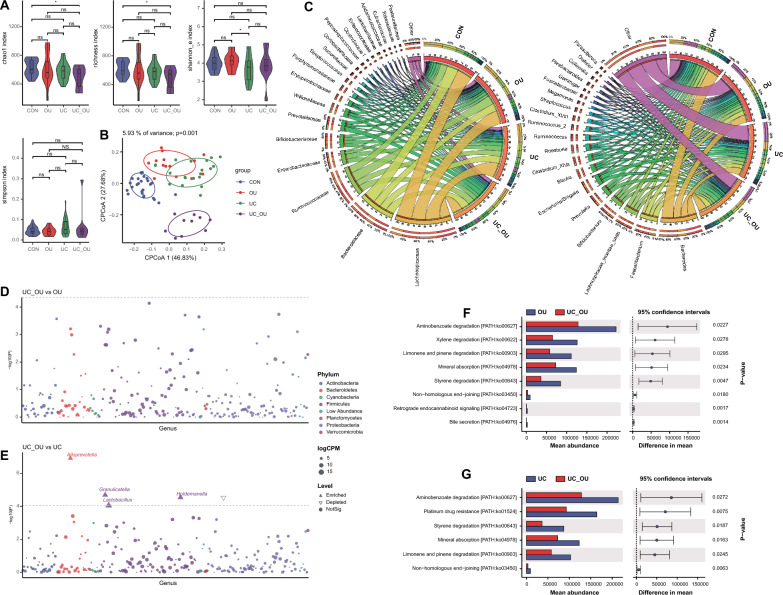
The bacterial profile in fecal samples of UC patients with or without oral ulcers. **A** Alpha diversity indices of the microbiota, including the richness, Simpson’s, Shannon’s, and Chao1 indices. Horizontal bars within boxes represent medians. The tops and bottoms of the boxes represent the 75th and 25th percentiles, respectively. The upper and lower whiskers cover 1.5 × the interquartile range from the upper and lower edges of the box, respectively. *P*-values were obtained using the one-way ANOVA test (comparisons among four groups). **B** The constrained principal coordinate analysis based on the Bray–Curtis distance. The R software (v 4.0.1) with the *vegan* (v 2.5–7) package were used, and *P*-values were obtained using permutational multivariate analysis of variance (PERMANOVA). **C**, Relative abundance of the top 20 bacterial families (the left panel) and genera (the right panel). Visualization was performed using Circos (http://circos.ca/). The right circle in the outer part shows the groups and relative proportions of bacterial species. The left outer circle and inner bands show the relative proportions (%) of bacterial genera in the different groups. The left inner circle represents the relative abundances of all bacteria. **D** and **E**, Comparative analysis of bacterial genus abundance between two groups (**D** UC_OU *vs.* OU; **E** UC_OU *vs.* UC). The EdgeR package was used for comparative analysis. The difference between the two groups is shown as a Manhattan diagram. Point shape indicates the genus enriched, depleted, or not significant in the former group compared with the latter. Point size indicates the counts of a specific genus. CPM, count per million. **F** and **G**, Comparative analysis of bacterial function between two groups [**F** UC_OU *vs.* OU; **G** UC_OU *vs.* UC]. Phylogenetic Investigation of Communities annotated the pathway information by Reconstruction of Unobserved States (PICRUSt2) software by referring to the Kyoto Encyclopedia of Genes and Genomes (KEGG) database. The STAMP software was used for data visualization. CON, healthy controls; OU, patients with only oral ulcers; UC, UC patients without oral ulcers; UC_OU, UC patients with oral ulcers; ns, not significant; *P-value < 0.05; **P-value < 0.01

We further analyzed the fecal bacterial composition of all subjects. We found that, compared with the CON, the alpha diversity of fecal bacteria in UC_OU patients significantly decreased, while the OU or UC patients did not (Fig. 4A and Additional file 5: Table S1); beta diversity analysis showed a marked difference in the fecal microbiota composition between patients and CON (Adonis *P* = 0.011 for OU, 0.002 for UC, and 0.003 for UC_OU *vs.* CON, respectively; Fig. 4B, S3A and Additional file 5: Table S2). In the top 20 bacterial families, Enterobacteriaceae increased in the groups of patients, while the abundances of Prevotellaceae in the UC and Veillonellaceae in the OU were decreased than in the CON group (Fig. 4C and Additional file 5: Table S5). At the genus level, compared with the CON, *Prevotella* and *Roseburia* declined in the UC group, while *Escherichia*/*Shigella* raised in the UC and UC_OU group (Fig. 4C and Additional file 5: Table S6).

We further performed a comparative analysis at the genus level. The results showed that, compared with the CON group, some bacteria (*e.g.*, *Arthrobacter*, *Knoellia*, *Bacillus*, *Peptostreptococcus, *etc*.*) increased in the groups of patients (OU/UC/UC_OU) and some decreased (*e.g.*, *Lautropia*) (Additional file 3: Figure S3B-D and Additional file 5: Table S11). Interestingly, *Bacillus* and *Peptostreptococcus* have been reported to correlate with severe infections [41] and IBD progression [42]. In addition, no differential genera could be found between UC_OU and OU groups (Fig. 4D); however, *Alloprevotella, Granulicatella*, *Lactobacillus,* and *Holdemanella* ascended strikingly in the UC_OU group compared with the UC group (Fig. 4E and Additional file 5: Table S11).

We then had the PICRUSt2 analysis. The results showed that some pathways, such as aminobenzoate degradation, xylene degradation, and bile secretion, lessened in the UC_OU patients compared with OU patients (Fig. 4F and Additional file 5: Table S12), among which xylene can exacerbate allergic inflammation [43]. Furthermore, the comparison between UC_OU patients and UC patients showed that metabolism-related pathways (including aminobenzoate degradation, platinum drug resistance, styrene degradation, etc*.*) were repressed (Fig. 4G and Additional file 5: Table S12). In addition, nucleotide metabolism and naphthalene degradation pathways in OU patients, and amino acid metabolism and fatty acid degradation in UC patients were activated compared with the CON (Additional file 3: Figure S3E-G and Additional file 5: Table S12)*.*

To conclude, the fecal flora characteristics in the UC_OU group were distinguished from UC alone, and *Alloprevotella*, *Granulicatella*, *Lactobacillus*, and *Holdemanella* may contribute to differentiating between UC_OU and UC alone. In addition, signal pathways related to metabolization and immunity can involve the pathogenic process in the UC_OU.

### The correlation of GI spatial microbiome with clinical, immunological parameters

**Fig. 5:**
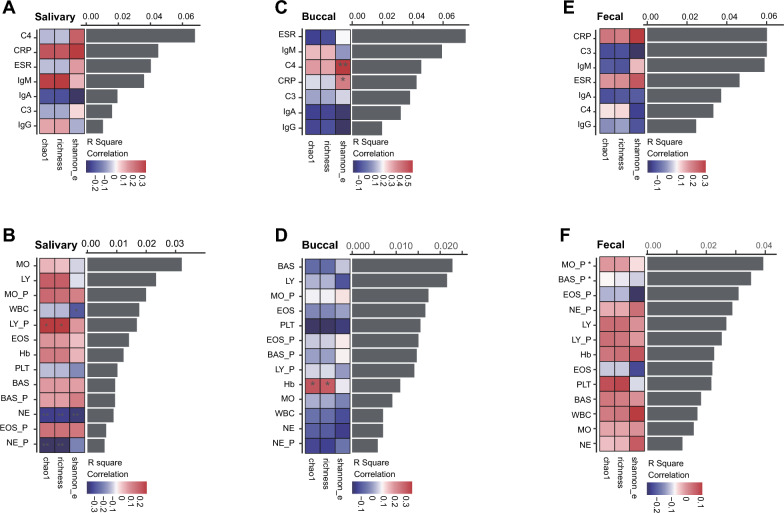
The correlation between clinical parameters (inflammatory and immunological) and bacterial diversity (alpha and beta). **A** and **B** The correlation between inflammatory (**A**) or immunological (**B**) indices and salivary bacterial diversity. The length of the black bar shows the R square value of the indices-beta diversity correlation. The results were acquired by the redundancy analysis (RDA) using the R software (v 4.0.1) with the *vegan* (v 2.5–7) package. The color box shows the *rho* value of indices-alpha diversity correlation. The results were analyzed from Spearman’s correlation using the R software (v 4.0.1) with the *vegan* (v 2.5–7) package. The star symbols behind the left words show the *P*-values acquired from the RDA. The box’s star symbols show the adjusted *P*-values (false discovery rate, FDR) received from the Spearman’s correlation. (same methodology as in **C**–**F**). **C** and **D** The correlation between inflammatory (**C**) or immunological (**D**) indices and buccal bacterial diversity. **E** and **F** The correlation between inflammatory (**E**) or immunological (**F**) indices and buccal bacterial diversity

To further assess whether the disease activities and clinical parameters correlated with the spatial microbiome alteration, we collected twenty clinical parameters responding to inflammatory and immunological statuses, including C-reaction protein (CRP), ESR, the percentage of monocytes (MO_P), etc*.* We performed a redundancy analysis (RDA) between bacterial beta diversity and clinical parameters and Spearman’s correlation between alpha diversity and clinical parameters (Fig. 5A–F and Additional file 5: Table S13). The inflammatory indices were statistically correlated with the salivary microbiome in alpha diversity and beta diversity (Fig. 5A). Of note, the salivary microbial alpha diversity correlated with the lymphocyte’s (*rho* = 0.281, *FDR* = 0.022 for chao1, and *rho* = 0.282, *FDR* = 0.022 for richness, respectively), and neutrophil’s percentage (*rho* = − 0.372, *FDR* = 0.002 for chao1, and *rho* = − 0.372, *FDR* = 0.002 for richness, respectively; Fig. 5B and Additional file 5: Table S13). The buccal bacterial Shannon index correlated with subjects’ inflammatory markers, such as C4 (*rho* = 0.567, *FDR* = 0.002) and CRP (*rho* = 0.399, *FDR* = 0.039) (Fig. 5C and Additional file 5: Table S13). Like the salivary microbiome, buccal bacterial beta diversity showed negative results correlating with clinical parameters (Figs. 5C, D and Additional file 5: Table S13). The fecal microbiota showed a significant correlation between beta diversity and immunological indices, such as the monocytes’ (*pseudo F* = 2.218, *P* = 0.039) and basophils’ percentage (*pseudo F* = 1.984, *P* = 0.035), rather than inflammatory markers (Fig. 5E, F, and Additional file 5: Table S13). Based on these results, we inferred that the fecal bacteria play a more important role in shaping the host immune system.

### Treatment responses in UC patients

**Fig. 6:**
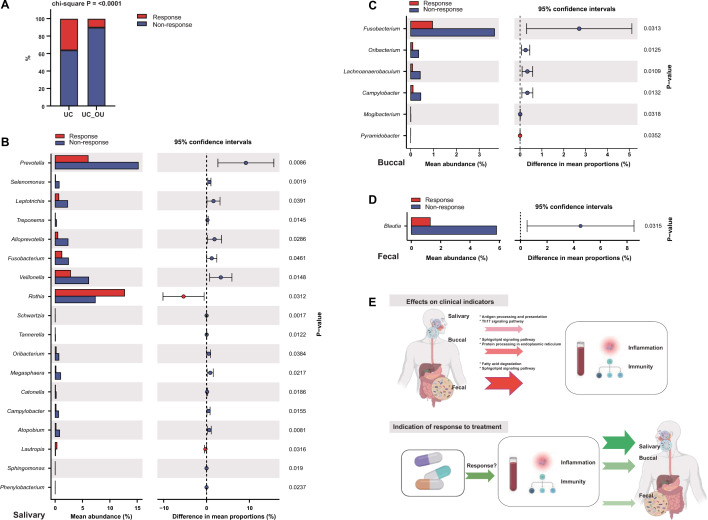
Microbial differentiation between UC patients with and without treatment response. **A** The treatment response rate in UC patients with or without oral ulcers. The *Chi*-squared test was performed to test the response difference between the two groups. UC, UC patients without oral ulcers; UC_OU, UC patients with oral ulcers. **B–D** The comparative analysis of bacterial genus abundance of the samples from salivary (**B**), buccal (**C**), and fecal (**D**) niches. The STAMP software was used for data visualization. **E** The graphic summary

We then analyzed the treatment response for UC patients with a six-month following-up. Thirty-two participants were followed-up, including twenty-two UC and ten UC_OU patients, for which we documented the Mayo clinic score to differentiate patients with or without treatment response. There were no significant difference of some clinical parameters (Mayo score, ESR, CRP, IgA, IgG, IgM, C3, and C4) before and after treatments in UC and UC_DU groups (Additional file 4: Figure S4). Interestingly, only one out of ten UC_OU patients responded to 5-ASA routine treatment, significantly lower than that in UC patients (*Chi-squared* test, *X*^*2*^ = 19.09, *P* < 0.001, Fig. 6A and Additional file 5: Table S14). We further analyzed the GI spatial bacterial profiles in UC patients with or without treatment responses. Only one subject had responded to the treatment in UC_OU patients, so this group of patients was excluded from this part of the investigation. Notably, there were eighteen genera with a remarkable difference in these two subgroups of patients in the salivary bacteria, including *Prevotella*, *Alloprevotella*, *Fusobacteria*, *Oribacterium*, *Campylobacter*, and *Rothia*, etc. (Fig. 6B and Additional file 5: Table S14). Compared with salivary samples, the buccal biopsies showed fewer bacterial contents, with a significant difference between UC patients with and without treatment responses (Fig. 6C and Additional file 5: Table S14). It could be found that there were three same genera also enriched in the non-responding UC patients’ oral mucosae, which were *Fusobacterium*, *Oribacterium*, and *Campylobacter*, respectively. Additionally, only one content, i.e., *Blautia*, was represented in fecal microbiota in non-responding patients (Fig. 6D and Additional file 5: Table S14). Based on these data, we deduced that the richness of *Fusobacterium*, *Oribacterium*, and *Campylobacter* might be involved in non-response; the salivary microbiome also had a potential for indicating treatment response in UC patients.

## Discussion

Previous studies indicate up to 34% of UC patients are accompanied by oral ulcers [8], while the microbial community composition of oral bacteria in patients with or without oral ulcers and the relationship between oral microbiota and IBD prognosis are still unclear.

This study found that each GI region had a different bacterial community, which consisting with previous study reported by Vasapolli [44]. Salivary samples and buccal samples shared more common and less unique bacteria, while fecal samples were richer in bacterial diversity and individual genera. These may be related to the distinct microenvironments [45], gastrointestinal acid [46], bile acid chemical barriers [47], intestinal microbial colonization resistance [48], host pattern recognition receptors [49], and loss of oxygen [50]. Moreover, compared with oral bacterial community, the fecal one was closely correlated with patient clinical parameters, especially the proportion of immune cells rather than inflammation-related indicators, suggesting that the fecal microbiota may modulate the function of the immune cells and participate in the pathogenesis of UC_OU patients. The abundance and part of the fecal bacteria may partially account for this phenomenon.

The variation in bacterial richness at different GI niches facilitates to differentiating UC_OU individuals from UC patients. Previous research has been proved that the *Klebsiella* isolated from the saliva of IBD patients colonizes in the intestine ectopically to elicit colitis development [51] and *Klebsiella*-reactive Th17 may migrate to the gut to exacerbate intestinal inflammation [52]. The *Blautia* and *Faecalibacterium* were treated as the functional genera with potential probiotic properties, for their metabolic regulation and butyrate-producing capacity [53, 54]. In our study, compared with UC patients, the *Klebsiella*, *Arthrobacter*, *Barnesiella* were enriched, and the *Blautia* and *Faecalibacterium* were depleted in the UC_OU patients’ oral samples, which indicates a more robust state of inflammation and immune activation in UC_OU patients.

The PICRUSt2 analysis indicated that the UC_OU group differs from the UC group and may be a new subclass of UC patients. Thiamine (also known as vitamin B1) is synthesized by bacteria and fungi and regulates the immune system by activating immune cells and proteins [55]. It is also an anti-inflammatory factor regulating inflammatory agent expression and preventing recurring inflammation [30]. Interestingly, Klaassen et al*.* have found that Crohn’s Disease exacerbations were associated with a decrease in microbial genes involved in the biosynthesis of the anti-inflammatory mediators, including thiamine [56]. Similarly, in our study, the reduction of thiamine metabolism in the salivary samples of UC_OU patients indicate a higher pro-inflammatory state than in UC patients. Furthermore, previous research found that sphingolipids are significantly decreased in IBD subjects and negatively correlated with gut inflammation [34]. The decreased glycosphingolipid biosynthesis in UC_OU patients’ buccal samples may imply more severe intestinal inflammation compared with the UC patients. Moreover, there was a positive correlation between styrene levels and disease activity in UC [57], and the suppressed styrene degradation pathway of fecal samples suggested higher styrene levels in UC_OU patients compared with UC. The functional analysis of the microbiota at the three sites suggests that the UC_OU group is different from the UC and may have a more severe inflammatory status.

We also compare the bacterial profiles at different GI spatial niches of treatment-non-responding UC patients. The genera, including *Fusobacterium*, *Oribacterium*, and *Campylobacter,* were enriched in the oral cavity of non-responding UC patients. Of note, these bacteria were also represented in patients with systemic lupus erythematosus (SLE) [58] and were involved in the intestinal inflammation of patients with distal gastrectomy [59]. A previous study reported that a *Fusobacterium* species, i.e.,* F. nucleatum*, in gut mucosa triggered gut inflammation and positively correlated with host IBD status [60, 61]. *Campylobacter spp.* was reported to recruit neutrophils to lead to gastroenteritis [62–64]. These data indicate that these oral bacteria might be related to therapeutic failure in UC patients. Of note, the salivary microbiome presented a markable difference in UC patients without response rather than the buccal or fecal microbiome. Additionally, a previous report confirmed that the oral microbes could recover to their initial state from collapsing in a few days and have sufficient robustness to serve as biomarkers [65, 66]. These results highlight the potential role of salivary microbiota as an indicator of IBD treatment efficiency.

## Conclusion

In conclusion, we analyzed the microbial community composition of the salivary, buccal and fecal samples of UC patients with or without oral ulcers by 16S rRNA sequencing and their correlation with clinical indicators. We found that UC patients with oral ulcers lacked treatment responses, and three oral bacterial genera might be involved. The fecal microbiota had the most significant impact on the host immune indices, and the salivary microbiota had the potential role in reflecting the treatment response of UC patients and is promising to be an indicator for UC treatment efficiency. Our data throw light on the significance of stratification of UC patients with oral diseases, and monitoring oral microbiota may be meaningful in assessing the therapeutic response in UC patients (Fig. 6E).

### Supplementary Information

**Additional file 1: Figure S1. **The comparative analysis of the salivary bacterial community. A, The constrained principal coordinate analysis (CPCoA) for three GI site samples based on the Bray-Curtis distance. The R software (v 4.0.1) with the *vegan* (v 2.5-7) package were used and *P*-values were obtained using permutational multivariate analysis of variance (PERMANOVA) (same methodology as B). B, The CPCoA for salivary samples based on the Bray-Curtis distance. C to E, The comparative analysis of salivary bacterial genus abundance between two groups ((C) OU *vs.* CON; (D) UC *vs.* CON; (E) UC_OU *vs.* CON). The *EdgeR* package was used for comparative analysis. The difference between the two groups is shown as a Manhattan diagram. Point shape indicates the genus enriched, depleted, or not significant in the former group compared with the latter. Point size indicates the counts of a specific genus. CPM, count per million. F to H, The comparative analysis of salivary bacterial function between two groups ((F) OU *vs.* CON; (G) UC *vs.* CON; (H) UC_OU *vs.* CON). Phylogenetic Investigation of Communities annotated the pathway information by Reconstruction of Unobserved States (PICRUSt2) software by referring to the Kyoto Encyclopedia of Genes and Genomes (KEGG) database. The STAMP software was used for data visualization. Abbreviations: CON, healthy controls; OU, patients with only oral ulcers; UC, UC patients without oral ulcers; UC_OU, UC patients with oral ulcers.

**Additional file 2: Figure S2. **The comparative analysis of the buccal bacterial community. A, The constrained principal coordinate analysis (CPCoA) for buccal samples based on the Bray-Curtis distance. The R software (v 4.0.1) with the *vegan* (v 2.5-7) package were used and *P*-values were obtained using permutational multivariate analysis of variance (PERMANOVA). B to D, The comparative analysis of buccal bacterial genus abundance between two groups ((B) OU *vs.* CON; (C) UC *vs.* CON; (D) UC_OU *vs.* CON). The *EdgeR* package was used for comparative analysis. The difference between the two groups is shown as a Manhattan diagram. Point shape indicates the genus enriched, depleted, or not significant in the former group compared with the latter. Point size indicates the counts of a specific genus. CPM, count per million. E to G, The comparative analysis of buccal bacterial function between two groups ((E) OU *vs.* CON; (F) UC *vs.* CON; (G) UC_OU *vs.* CON). Phylogenetic Investigation of Communities annotated the pathway information by Reconstruction of Unobserved States (PICRUSt2) software by referring to the Kyoto Encyclopedia of Genes and Genomes (KEGG) database. The STAMP software was used for data visualization. Abbreviations: CON, healthy controls; OU, patients with only oral ulcers; UC, UC patients without oral ulcers; UC_OU, UC patients with oral ulcers.

**Additional file 3: Figure S3. **The comparative analysis of the fecal bacterial community. A, The constrained principal coordinate analysis (CPCoA) for fecal samples based on the Bray-Curtis distance. The R software (v 4.0.1) with the *vegan* (v 2.5-7) package were used and *P*-values were obtained using permutational multivariate analysis of variance (PERMANOVA). B to D, The comparative analysis of fecal bacterial genus abundance between two groups ((B) OU *vs.* CON; (C) UC *vs.* CON; (D) UC_OU *vs.* CON). The *EdgeR* package was used for comparative analysis. The difference between the two groups is shown as a Manhattan diagram. Point shape indicates the genus enriched, depleted, or not significant in the former group compared with the latter. Point size indicates the counts of a specific genus. CPM, count per million. E to G, The comparative analysis of bacterial function between two groups ((E) OU *vs.* CON; (F) UC *vs.* CON; (G) UC_OU *vs.* CON). Phylogenetic Investigation of Communities annotated the pathway information by Reconstruction of Unobserved States (PICRUSt2) software by referring to the Kyoto Encyclopedia of Genes and Genomes (KEGG) database. The STAMP software was used for data visualization. Abbreviations: CON, healthy controls; OU, patients with only oral ulcers; UC, UC patients without oral ulcers; UC_OU, UC patients with oral ulcers.

**Additional file 4: Figure S4. **The alteration of clinical parameters from baseline (Before) to the end of follow-up in UC patients with or without oral ulcers. A, The mayo clinic score. B, Erythrocyte sedimentation rate (ESR); C, C-reaction protein (CRP); D, Immunoglobulin A (IgA); E, Immunoglobulin G (IgG); F, Immunoglobulin G (IgG); G, Complement 3 (C3); H, Complement 4 (C4).

**Additional file 5: Supplementary tables.** **Table S1.** Bacterial alpha diversity of each sample. **Table S2.** Bray Curtis distance of each sample. **Table S3.** Shared bacterial families and genera in three different GI niches. **Table S4.** Specific bacterial abundance for the ternary plot. **Table S5.** Relative bacterial family abundance of each sample. **Table S6.** Relative bacterial genus abundance of each sample. **Table S7.** Comparative analysis for bacterial community in the salivary samples (all results presented). **Table S8.** Comparative analysis for bio-function in the salivary samples (only significant results presented). **Table S9.** Comparative analysis for bacterial community in the buccal samples (all results presented). **Table S10.** Comparative analysis for bio-function in the buccal samples (only significant results presented). **Table S11.** Comparative analysis for bacterial community in the fecal samples (all results presented). **Table S12.** Comparative analysis for bio-function in the fecal samples (only significant results presented). **Table S13.** Correlative analysis between clinical parameters (inflammatory and immunological) and bacterial diversity (alpha and beta). **Table S14.** Bacterial comparative analysis of UC patients with or without treatment response at three different GI niches.

## Data Availability

The 16S rRNA-seq data have been deposited in the Genome Sequence Archive in National Genomics Data Center, China National Center for Bioinformation / Beijing Institute of Genomics, Chinese Academy of Sciences (GSA: CRA006292) that are publicly accessible at https://ngdc.cncb.ac.cn/gsa/. The data supporting the findings of this study are available within the article and its Supplementary Information files.

## Abbreviations

C3: Complement C3
C4: Complement C4
CRP: C-reactive protein
ESR: Erythrocyte sedimentation rate
IgM: Immunoglobulin M
IgA: Immunoglobulin A
IgG: Immunoglobulin G
MO: Monocyte
LY: Lymphocyte
MO_P: Monocytes’ percentage
WBC: White blood cell
LY_P: Lymphocyte’ percentage
EOS: Eosinophil
Hb: Hemoglobin
PLT: Platelet
BAS: Basophils
BAS_P: Basophils’ percentage
NE: Neutrophils
NE_P: Neutrophils’ percentage
ASV: Amplicon sequence variants
COPD: Chronic obstructive pulmonary disease
CPCoA: Constrained Principal Co-ordinates Analysis
FDR: False discovery rate
FMT: Fecal microbiota transplantation
IBD: Inflammatory bowel disease
MAFLD: Metabolic associated fatty liver disease
PERMANOVA: Permutational multivariate analysis of variance
RDP: Ribosomal Database Project
PICRUSt2: Phylogenetic Investigation of Communities annotated the pathway information by Reconstruction of Unobserved States
KEGG: Kyoto Encyclopedia of Genes and Genomes
